# Blood Sampling in Göttingen Minipigs—A Case Study of Two Standard Methods and Clicker Training as a Restraint-Free Alternative

**DOI:** 10.3390/ani15030407

**Published:** 2025-02-01

**Authors:** Kathrine Schiøler, Mikkel Lykke Jensen, Dorte Bratbo Sørensen

**Affiliations:** 1 Department of Veterinary and Animal Sciences, Faculty of Health and Medical Sciences, University of Copenhagen, Gronnegaardsvej 15, 1870 Frederiksberg C, Denmark; 2 Scantox A/S, Hestehavevej 36A, 4623 Lille Skensved, Denmark; mlj@scantox.com

**Keywords:** animal welfare, laboratory minipigs, blood sampling, saliva sampling, clicker training

## Abstract

Blood sampling often requires restraint that negatively affects animal welfare. This case study evaluated three methods for jugular vein blood sampling (V-bench, sling, and clicker training) with the overall aim of assessing the negative impact on animal welfare and providing a general recommendation on the use of methods. First, the effects of simulated blood sampling in V-bench and sling on behaviour, heart rate, and saliva cortisol concentrations 15 min after sampling were assessed. The sling (which had the least negative effects) was re-evaluated after one week of habituation to assess a possible positive effect. Clicker training was evaluated using behavioural observations only. The heart rate was significantly higher in animals placed in the V-bench compared to the sling (*p* < 0.05), but no significant differences were found in the saliva cortisol concentrations. Habituation to the sling resulted in a further reduction in heart rate compared to the V-bench (*p* < 0.01). When trained to voluntarily cooperate, three out of six minipigs allowed blood sampling from the jugular vein without restraint. We are convinced that all six minipigs would have succeeded if the training environment had been optimised. In conclusion, clicker training results in blood sampling with no negative behaviours displayed such as struggling or squealing/screaming. When restraint is unavoidable, the sling is preferable compared to the V-bench.

## Simple Summary

This study focuses on minimising stress and discomfort in laboratory minipigs during blood sampling procedures. We assessed three methods for jugular vein blood sampling: two methods that require restraint (the V-bench and the sling), and clicker training, where the pigs are trained to voluntarily cooperate during the procedure. The goal was to evaluate how these methods impact animal welfare and recommend the best approach. The study examined the effects of simulated blood sampling in the V-bench and the sling on behaviour, heart rate, and saliva cortisol levels 15 min after sampling. Overall, the sling method showed fewer negative effects on the animals compared to the V-bench. Clicker training allowed three animals to voluntarily cooperate; however, we firmly believe all six could have succeeded in a better training setup. In summary, clicker training results in blood sampling with no negative behaviours such as struggling or squealing/screaming. When restraint is unavoidable, the sling is a better alternative to the V-bench. By identifying methods that allow animals to voluntarily cooperate, a higher standard for animal care is made available, improving both the lives of animals and the quality of data obtained for scientific and medical purposes.

## 1. Introduction

Experimental animals such as pigs are often subjected to environmental stressors relating to housing, handling, and experimental procedures. These conditions may compromise the welfare of the animals [1,2,3] and affect the production of corticosteroids and adrenalin, resulting in changes in, for example, the animal’s metabolism, immune function, heart rate, and blood pressure [4,5]. Such changes could impact the study results, thereby influencing the study’s outcome and hence its reproducibility [2,4,6]. When working with laboratory animals, adherence to current legislation and 3R (Replacement, Reduction, and Refinement) without compromising the research’s scientific or medical aims is essential [7,8,9,10], and medical procedures such as blood sampling should continually be Refined to ensure that these interventions cause minimal strain on the animal.

Blood sampling in laboratory minipigs is a stressful procedure, especially in studies that demand multiple samples a day and restraint is often considered necessary to ensure the safety of the minipig and personnel during sampling. One of the standard blood sampling methods requires the minipig to be restrained in dorsal recumbency on a V-bench, a position that is seldom tolerated by the animals. As a refinement, Ellegaard Göttingen Minipigs A/S in Denmark introduced the collecting of blood samples while minipigs were placed in a sling. The use of the sling reduced vocalisation during restraint indicating a lower level of stress and fear in the pigs [11]; however, these results should be confirmed by the use of physiological parameters, such as saliva cortisol concentrations and heart rate. Additionally, the study by Christoffersen et al. (2011) at Ellegaard Göttingen Minipigs did not assess if the use of the sling can be further improved by performing repeated presentations of the restraint scenario combined with food rewards prior to the study-related samplings, which could represent a possible refinement of the method. These repeated presentations are often performed with the purpose of familiarising the animal with the procedure, and in the field of laboratory animals, this approach is traditionally referred to as habituation. It is questionable whether such an approach is, in fact, habituation [12,13], but this discussion is outside the scope of this paper.

Hypothesising that clicker training (CT) would represent an even better alternative to both the V-bench and sling, even after habituation, we proceeded with clicker training of the pigs. Training for voluntary blood sampling has been successfully used in laboratory primates and zoo animals for decades [14,15], and increasing interest is seen in other laboratory animal species such as rodents [16,17]. Minipigs are highly trainable [18], and training minipigs to cooperate and voluntarily participate in the procedure using positive reinforcement would most likely result in reduced stress and enhanced refinement [14,19,20,21]. Laboratory minipigs have previously been clicker-trained to, for example, follow a target, exhale into an expiration bag, accept injections, or freeze during the collection of multiple X-rays [20,22], but no studies have—to our knowledge—been published on laboratory minipigs giving assent to blood sampling. We aimed to demonstrate that it was possible to train the pigs despite the pigs’ previous history of being restrained and that training in voluntary blood sampling could be achieved within an acceptable time frame.

Physiological stress was assessed by measuring cortisol concentrations in saliva and heart rate [23,24,25,26,27,28,29]. Additionally, stress in minipigs can be assessed by observing high-frequency, open-mouth vocalisation [30] and struggling during the procedures [31]. In unpublished pilot studies, we have shown that clicker-trained pigs do not react to blood sampling from the jugular vein using a single-use 21 G needle and a Vacuette^®^. A logical consequence is that it is primarily the restraint that untrained pigs react strongly to. Additionally, it is known that stress related to, for example, acute or chronic restraint or fixation can give rise to increased pain sensitivity in both humans and non-human species [32,33], which can also be avoided if the pigs participate voluntarily.

This study aimed to (1) confirm that the use of the sling is less stressful compared to the V-bench; (2) assess whether habituation to the sling would further reduce the negative impact on animal welfare (assuming that the sling was the better alternative); and (3) demonstrate that clicker-training was a force-free alternative to both the sling and the v-bench-aided blood sampling.

## 2. Materials and Methods

### 2.1. Animals and Housing 

Six Göttingen minipigs (Ellegaard Göttingen Minipigs A/S, 4261 Dalmose, Denmark) were included in this study. The minipigs were aged between 6 and 11 months and weighed 14 to 22 kg. They were housed at Scantox A/S, Ejby, Denmark. The pigs had different past experiences with various procedures, as they had previously been used by the animal staff for practising various procedures. One pig (pig number 5) had previously been used in a toxicology study (Table 1). From a 3R perspective, it was decided to use these pigs instead of purchasing new ones. All pigs had been given a minimum of two weeks with no procedures before entering this study. All pigs were tested both in the V-bench and the sling and were also clicker trained. None of the pigs had previously been clicker-trained or otherwise trained in the procedures.

**Figure 1:**
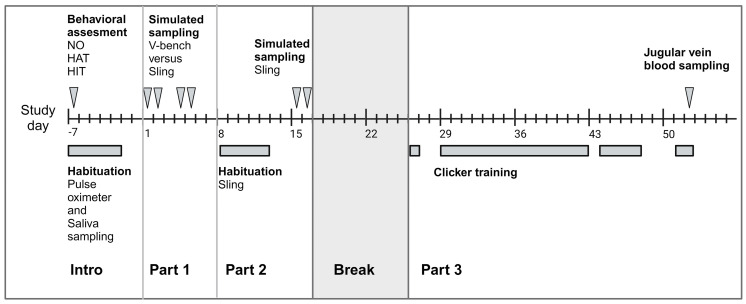
Timeline for the study including the first week of behavioural assessment and habituation to pulse oximeter and saliva sampling (week −1). Day 1 was considered the start of the study. NO: novel object test. HAT: human approach test. HIT: human interaction test.

The gilts were group-housed in a double-pen (6 m^2^) with a partition wall and a door, so they could be separated if necessary. All the gilts were familiar with being separated for up to three hours. The boars were housed individually in the same animal room with the gilts. The temperature in the animal room was 21 °C ± 3 K with a minimum of 10 air changes per hour. The light was on from 06:00 h to 18:00 h. The bedding in the pens was woodchips (Aspen bedding 2HV; Tapvei Estonia Oü, 75408 Harjuma, Estonia). The pens were cleaned once daily. All pens were supplied with toys already used previously such as Kong Toys (red, XL, certified) and Megalast balls (large, certified). All enrichment items were supplied by Bio-serv (Flemington, NJ 08822, USA). Toys placed on the floor were replaced with a different, clean toy after cleaning the pen. When available, large paper bags or cardboard boxes were also used as enrichment. At least once weekly, enrichment in the form of ice cubes with food pellets or frozen acidified milk was provided to the pigs. Further, pigs were given a daily supply of grass straw (dehydrated Compact Gras, Hartog B.V., 1658 CA Lambertschaag, the Netherlands) to allow the animals to display rooting behaviour. All minipigs were fed 125 g extrudate twice daily (Altromin 9069; Breeding & Maintenance diet for minipigs; Altromin Specialfutter GmbH & Co. KG, 32791 Lage, Germany) and tap water ad libitum. The minipigs were weighed every other week and checked for any clinical signs of illness daily. The study lasted 59 days (see Figure 1). Parts 1 and 2 of the study focussed on the restraining methods (V-bench and sling) and were conducted with simulated blood samplings to minimise the negative impact on the animals. For the clicker training part of the study (part 3), blood samples were drawn from the jugular vein to verify that the minipigs participated voluntarily and without hesitation in the blood sampling procedure.

### 2.2. Behavioural Assessment (Intro)

As the minipigs had different training histories, a simple, initial behaviour assessment was conducted prior to the start of the study to ensure that all minipigs were well-socialised and comfortable in their home pen. To assess their level of curiosity, a novel object (NO) test was conducted. A colourful bucket was placed inside their pen, and the latency to approach the bucket was recorded. The test was modified from Haagensen et al. (2014) to home pen use [34]. Following the novel object test, a simple human approach test (HAT) measuring the latency to approach [35] and a human interaction test (HIT) were performed to assess the level of socialisation. An unknown technician entered the pen and stood still next to the bucket. The latency to approach the technician was recorded. After 60 s, the technician extended her hand towards the pig, and the time taken by the pig to calmly accept being touched behind its ears was recorded. As expected, it was concluded that all pigs were appropriately socialised, and no further socialisation was necessary (Supplemental Figure S1).

### 2.3. Habituation to Saliva Sampling and Heart Rate Measurement

In week −1 of the study (Intro from day −7 to day −3), all minipigs were habituated to the Salivette^®^ saliva sampling method and the Nonin^®^ pulse oximeter (Nonin Medical B.V., Tilburg, The Netherlands). Each day, the minipigs were connected to the pulse oximeter by an ear clip for at least 2 min, and the heart rate was noted. Afterwards, all minipigs were introduced to the saliva sampling method. The cotton swab from the Salivette^®^ tube (Sarstedt AG & Co. KG, 51582 Nümbrecht, Germany) was attached to a clean haemostat and gently, without force, inserted into the minipig’s mouth. They were then allowed to chew on the cotton swab for 1–2 min. After five days, all minipigs were calm during the pulse oximeter recording. Except for pig nr. 5, all pigs willingly engaged in the saliva sampling and readily took the cotton swab and chewed until the technician, with slight force, retracted the cotton swab. Pig number 5 was more hesitant, and the technician had to gently force the cotton swab into the mouth before the pig began to chew on it.

### 2.4. Simulated Blood Sampling Procedure in V-Bench and Sling (Part 1)

The minipigs were allocated into two groups to ensure that saliva samples for cortisol analysis were collected over a short period of time as cortisol levels show a clear circadian rhythm [36]. Each group consisted of three minipigs, weight-stratified and with both genders represented. The group number determined which day they were tested (Table 2). The minipigs were placed in a V-bench and a sling for a simulated blood sampling procedure using a pen to simulate a cannula (see Figure S2). The V-bench was a V-shaped, cushioned restrainer in which the pig was placed in dorsal recumbency. The sling was a modified, soft sling with a soft mandibular support where the pigs were placed in sternal recumbency (see Figure S2). The pigs were restrained on different days with a rest period of two days between the two simulated samplings. Since the minipigs differed in size and bodyweight, they required different sizes of slings. To avoid changing slings during the test days, four out of six minipigs started with a simulated blood sampling in the V-bench, while two out of six minipigs started in the sling (Table 2). Vocalisations and escape-related behaviour (struggling) were noted on all test days 1–2 and 4–5 (Table 3).

### 2.5. Sling Habituation and Simulated Blood Sampling (Part 2)

On days 8–12, all minipigs were habituated to the sling. Each minipig was placed in the sling for about 10 s and then rewarded with food pellets or apple juice after being returned to their pen. In week 3 (test days 15 and 16), after one week of habituation to the sling, all minipigs were placed in the sling for a simulated blood sampling procedure (Table 2).

### 2.6. Saliva Sampling for Cortisol Measures

The cortisol concentration in pigs follows a daily rhythm and concentration is highest shortly after awakening in the morning and then decreases during the day. As saliva cortisol levels are an ultrafiltrate of the free steroid fraction in the blood, this circadian rhythm is also reflected in the cortisol concentrations in saliva and must be accounted for during sampling [23,24,36] scri. Another point of attention is that saliva cortisol concentrations peak 15–30 min after the stressful event. Traditionally, increases in cortisol levels are related to aversive events such as handling, blood sampling, environmental challenges, social stress and transport stress [26,37,38]. However, increases in cortisol levels may also be found during positive social encounters such as reunion with a group [39]. It should also be noted that the pigs in this study differed in gender and age, which may also result in different plasma concentrations of cortisol [40].

On each test day (study days 1, 2, 4, 5, 15, and 16), saliva samples for baseline cortisol levels (TB; time baseline) were collected between 8:30 and 9:00 in the morning, which was at least 1 h after the pigs’ morning feeding. Between 9:05 and 9:25, all three pigs from either Group 1 or 2 were placed in either the V-bench or the sling for their simulated blood sampling procedure. A pen was gently pressed against the ventral part of the neck to simulate a blood sampling from the external jugular vein. While placed in their respective restraining method, the minipig’s heart rate was measured, and a saliva sampling to the timepoint of 0 min was collected (T0). Hereafter, the minipigs were placed in their pens again, and saliva samplings to the timepoints 15 min, 30 min, and 60 min were collected under calm conditions (T15, T30, and T60). Therefore, all 15 saliva samplings were collected between 08:30 and 10:25 in the morning on each test day.

The Salivette^®^ tubes were stored in ice water while saliva samplings were collected. All 15 samples were centrifuged at 2200 RCF at 4 degrees for 10 min. Between 250 µL and 750 µL clean saliva was pipetted to 1.8 mL Nunc^®^ Cryogenic tubes. The samples were then stored at −80 degrees Celsius until further analysis.

### 2.7. ELISA Analysis

A competitive ELISA kit (Cortisol ELISA, IBL International, Hamburg, Germany), previously validated for pigs’ saliva, was used to determine the cortisol concentration in the samples [41]. The assays were conducted as described in the protocol provided by the +manufacturer (IBL International GmbH, Germany). The plates were shaken for 2 h on an orbital shaker (New Brunswick Innova^®^ 40/40R, Benchtop Orbital shaker (New Brunswick Scientific Co Inc., Edison, NJ, USA)). An automatic plate washer was used for the washing procedure (Thermo Scientific^®^, Wellwash^®^ Microplate Washer (Thermo Fischer Scientific Oy, FI-01621 Vantaa, Finland)). At the end of the ELISA procedure, the optical density was measured by a Tecan Sunrise^®^ absorbance microplate reader (Tecan Austria GmbH, A-5082 Grödig/Salzburg, Austria). Four-parameter logistics were applied as recommended for the calculations. Cortisol concentrations were measured in ng/mL.

### 2.8. Heart Rate Measure

The minipig’s heart rate (beats per minute) [42] was measured using a Nonin^®^ pulse oximeter with an ear clip. All pigs had previously been introduced to the device (see Section 2.3). The heart rate measurement was performed after the simulated blood sampling, while the pigs were still placed in their respective restraining device.

### 2.9. Clicker Training (Part 3)

The clicker training was initiated on study day 26 and the minipigs were trained using positive reinforcement for 21 days, with a maximum of three consecutive days without training during the five weeks (see Figure 1). From training day 5, the minipigs were given the opportunity to walk freely around in the hallway and the room at the back of the stable and explore the surrounding environment for about 5–10 min each day before the training sessions started.

Clicker training builds on the operant conditioning principle known as positive reinforcement combined with the use of a conditioned reinforcer, in this case a clicker [43,44,45]. In short, the trainer initially conditioned the clicker by presenting the “click” sound immediately followed by the reinforcer (unconditioned). In this study, apple juice delivered by the trainer from a plastic squeeze bottle was used. After a few trials, the pig associated the sound of the clicker with the paired reinforcer, and the clicker became the conditioned stimulus predicting the reinforcer (the apple juice). The clicker thereby bridged the desired behaviour and the reinforcer, which allowed the trainer to mark the desired behaviour with accuracy and subsequently give the pig the reinforcer. The target stick consisted of a metal bar with a clicker attached to one end and a green rubber ball attached to the other (Supplemental Video S1). The green ball was the target; each time the minipigs touched the ball with their snout, the trainer “clicked” and immediately offered a sip of apple juice from the squeeze bottle. When “follow target” was trained, the trainer clicked for following, but not touching, the target. A click was always followed by the reinforcer (the apple juice) to ensure that the association between the reinforcer and the conditioned reinforcer remained stable. The wagon consisted of various-sized red plastic plates that formed the wagon’s sides, a metal frame to hold the entire structure, and a Krokfjorden bathroom shelf from IKEA^®^ as a snout station. The metal frame was placed on top of a mobile, adjustable lift table with a ramp attached to make the fully assembled wagon (Supplemental Video S1). From training step 7 forward, at least one representative training session was video-documented daily (not included).

Each minipig experienced 10 clicks followed by apple juice per training session and two sessions per day (a total of 20 clicks per day). They were trained individually for five minutes daily during the first seven days of training and two minutes per day in the remaining fourteen days. The minipigs experienced a total of approximately 60 min of positive reinforcement training during this study. Within 21 days, the minipigs learned the 13 steps described in the training protocol (Table S1). It should be noted that during the training, the minipigs could always choose to return to their pen and end their training session for the day as they were not physically restrained in any way. If a pig chose to end the training session by returning to the pen, the trainer would follow and give the pig a sip of apple juice in the pen and end the training session formally. In this way, the pigs had a genuine choice as both continuing the training as well as ending the training resulted in apple juice. In other words, if a pig chose to end the training by returning to the pen, this behaviour was not punished by the trainer.

The complete, final behaviour aimed for was this: the minipig follows the target stick up the ramp and into the wagon, puts the snout on station and remains calm while a blood sample is drawn from the jugular vein. Hence, the complete behavioural sequence consisted of several behavioural elements: (1) following the target stick, (2) standing calmly on the station, (3) walking on novel surfaces, (4) walking on a slope, (5) entering a “cage-like” wagon, and (6) accept being touched and pinched on the neck.

All these behavioural elements were trained using shaping, i.e., reinforcing small steps (successive approximations) towards the desired behaviour. When fluent, they were gradually combined to create the overall behavioural sequence: the cooperative blood sampling behaviour. The more difficult behavioural elements were largely trained first, so for example “walking on a novel surface” was trained and maintained prior to “accepting being touched on the neck”, which was an easy behaviour to present for the well-socialised minipigs. By doing this, the reinforcement history would be longer for the more difficult behavioural elements (e.g., “walking on a novel surface”) of the final sequence, thus allowing more time to reach fluency.

The order in which to train the various behavioural elements may of course differ from case to case depending on the animals’ training history. In our study, all these behavioural elements were trained in the same order in all the pigs.

The final blood sampling on day 52 was performed using a vacutainer system mounted with a single-use 21 G needle (21 G × 1 1/2” Vacuette^®^ multiple use drawing needle, ref 450076. Greiner Bio-One GmbH, 4550 Kremsmünster, Austria).

### 2.10. Data and Statistics

The mean concentrations from time points T0, T15, T30, and T60 were compared to the mean baseline concentrations (TB) for each of the three simulated blood samplings. The saliva cortisol concentration data were not normally distributed and nonparametric statistics, in this case Friedman’s test followed by Dunn’s multiple comparisons test, were applied. Saliva sample T0 min from minipig number 4 was mistakenly first collected in the pen. However, since the saliva cortisol concentrations first peak around 15–30 min after the stressful event [23], this sample was still included in the statistics. T0 from minipig number 2 on the day he was placed in the sling was removed from the statistics because it was an outlier.

The data for the heart rate measurements were all normally distributed; hence, the two-way ANOVA with repeated measurements and Tukey’s multiple comparisons test were applied. Due to a deviation from the study protocol, heart rate measurement from minipig number 4 during blood sample simulation in the V-bench was removed from the dataset.

All *p*-values < 0.05 were considered significant. The statistical computer program GraphPad Prism 10 was used to calculate all the statistics.

## 3. Results

### 3.1. Behavioural Assessment During Simulated Blood Samplings in V-Bench and Sling

All minipigs squealed the moment they were captured in their pen, lifted from the ground, and carried by the technician (Before = B; Table 4). Four out of six minipigs vocalised while being restrained in the sling, while all six minipigs vocalised while being restrained in the V-bench (During = D, Table 4). Four out of six minipigs vocalised while being lifted back into their pen after being restrained in both the sling and the V-bench (After = A, Table 4). Only one minipig struggled while being captured and lifted into both of the restraining methods. Two minipigs struggled while being restrained in the V-bench—three did while being restrained in the sling. Lastly, three minipigs struggled while being lifted back into their pens after both restraining methods (Table 4).

### 3.2. Sling Habituation

After five days of habituation (days 8 to 12), all minipigs except number 4 were calm in the sling for over 20 s. Number 4 was calm in the sling for 5 s.

### 3.3. Saliva Cortisol Concentrations

**Figure 2:**
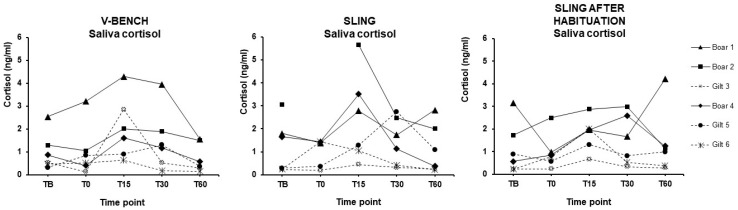
Saliva cortisol concentrations of individual animals from five time points for each of the three simulated blood sampling procedures (V-bench, sling, and Sling after Habituation).

**Figure 3:**
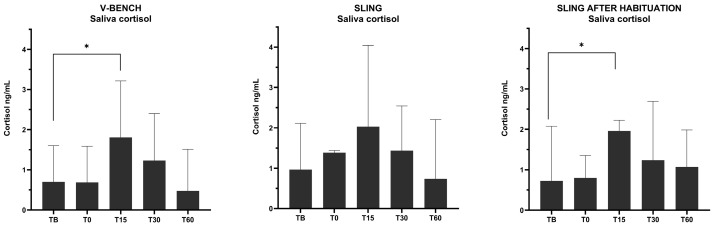
Saliva cortisol concentrations (median and Interquartile range (IQR)) from the five time points for each of the three simulated blood sampling procedures (n = 5 in sling T0, n = 6 at all other time points) (* = *p* ≤ 0.05).

The saliva cortisol concentrations are presented in Figure 2 and Figure 3. T15 was significantly higher in the V-bench (*p* = 0.0349) and Sling after Habituation (*p* = 0.0423) compared to their respective TB (Figure 3). There was a tendency towards a difference between TB and T15 in the sling (*p* = 0.0760).

There were no significant differences between the three TB concentrations. In addition, there were no significant differences between the means of the three T15 concentrations indicating they might induce the same level of stress response. Notably, the Interquartile range (Q3–Q1) from the T15 concentration in the Sling after Habituation (IQR = 1.08) seemed to be lowered compared to the sling (IQR = 3.15) and V-bench (IQR = 2.38).

### 3.4. Heart Rate Measurements

**Figure 4:**
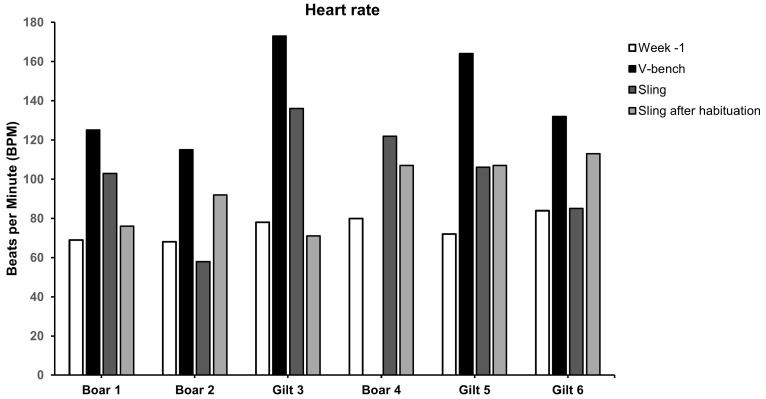
Individual heart rates as Beats per Minute (BPM) for all six minipigs as baseline (TB) and during simulated blood sampling in V-Bench, sling and Sling after habituation.

**Figure 5:**
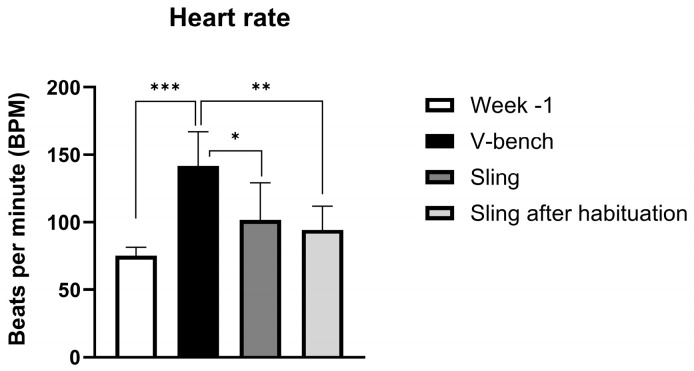
Heart rate as Beats per Minute (BPM; mean +/− SD). All heart rate measurements from week −1 (baseline, TB) and all three simulated blood sampling procedures (n = 5 in V-Bench, n = 6 at all other procedures) (* = *p* ≤ 0.05, ** = *p* ≤ 0.01, *** = *p* ≤ 0.001).

The heart rate was significantly higher in animals placed in the V-bench compared to the sling and Sling after Habituation (142 ± 26 BPM vs. 102 ± 28 and 95 ± 18 BPM, *p* < 0.05 and *p* < 0.01, respectively (see Figure 4 and Figure 5)). There were no significant differences between the average heart rates from week −1 and those measured in the sling or the Sling after Habituation (see Figure 5).

### 3.5. Clicker Training

Males number 2 and 4 directed most of their behaviour towards the gilts in estrus and showed no interest in apple juice. It was therefore not possible to train these boars outside their home pen. Since this study had to follow a strict schedule, we decided to exclude boars number 2 and 4 from the remaining part of the training. Gilt number 6 did not reach the final criteria (criteria 13, Table S1). Jugular blood samples with no restraint were successfully drawn from gilts number 3 and 5 on day 21 (Video S1). Boar number 1 also allowed the needle to be inserted three times, but the technician failed to locate the jugular vein. No pigs displayed open-mouth vocalising (squealing) during their training. Oinks were heard occasionally.

## 4. Discussion

The first part of this study aimed to determine if the sling was a less stressful blood sampling procedure than the V-bench. The saliva cortisol concentrations 15 min after the simulated blood sampling procedure were significantly increased in the V-bench group. This is in agreement with a study by Cerón et al. (2022), which concluded that peak saliva cortisol concentration will be observed 15 to 30 min after a stressful event [24]. The sling group did not differ significantly in T15 saliva concentration compared to the baseline. This finding is in accordance with the study by Puy et al. (2024) [27]. However, there was a tendency towards a difference between TB and T15 in sling (*p* = 0.0760). The results from the six minipigs during their simulated blood sampling procedures should, however, be interpreted cautiously as the variation is high.

In part 2, the median saliva cortisol concentration at T15, as well as the IQRs, were not significantly reduced by one week of habituation. Interestingly, the Interquartile range (Q3–Q1) from the T15 concentration in the Sling after Habituation (IQR = 1.08) seemed to be lowered compared to the sling (IQR = 3.15) and V-bench (IQR = 2.38). This finding should be interpreted with caution, but it could indicate that habituation to the sling may reduce variation, which calls for further studies [28]. Comparing all three samplings (V-bench, sling and Sling after Habituation), no significant differences between the three saliva cortisol-means at T15 were found, indicating that all three methods may be equally stressful for the laboratory minipigs.

Blackshaw and Blackshaw (1989) stated that saliva cortisol shows a lack of correlation with plasma levels [46]. However, other studies indicate that saliva cortisol correlates well with plasma concentrations although a 10–15 min delay is found ([26,47,48]. This delay results in difficulties discerning the stress of the restraining method per se from the stress induced by the handling (capturing, lifting, and carrying of the pigs) prior to the restraining. On the other hand, this may be of limited importance when testing classical set-ups such as the V-bench or the sling, as it is not possible to perform any of these blood sampling procedures without capturing and handling the pigs. On every test day during V-bench and sling testing, all minipigs vocalised when they were approached and lifted by the technician, even though all minipigs were adequately socialised prior to the beginning of the study. These behavioural observations indicate that the pigs started responding negatively when approached and captured in their pen. As all minipigs responded with vocalisation in this process, it is not surprising that they had a rise in their cortisol concentration 15 min later.

Heart rate measurements indicated in real-time the minipig’s reaction while placed in the sling or V-bench. However, the heart rate will most likely also be affected by events prior to simulated blood sampling. As handling and restraint are integral parts of the procedures, a more precise measure separating the effect of handling from restraint and simulated blood sampling would require the use of a heart-rate sensor such as the Polar Equine Heart Rate Monitor (Polar Electro Oy, 90440 Kempele, Finland)) placed on the pigs using a strap or a belt. This method was unfortunately not available in the present study, but it would be advisable to use this approach if further studies are planned. Using such a device would also allow for measuring, for example, the heart rate variability (HRV), which could provide a more detailed assessment of the stress level of the pigs [49,50]. The results from this study seem to indicate that minipigs were more stressed in the V-bench than in the sling. There were no significant differences between the minipigs’ average heart rate measures at the end of the habituation to the pulse oximeter (week −1) and their heart rates when placed in the sling. This could indicate that the significant increase in cortisol at T15 from the sling may result from the minipig’s handling and placement rather than the time the minipig spends in the sling. This could also be the case regarding the significantly increased T15 in the V-bench. Regardless, the minipig’s heart rates were significantly increased in the V-bench compared to baseline values and when in the sling, indicating that blood sampling in the sling would be less stressful for laboratory minipigs compared to the V-bench. Interestingly, the Interquartile range (Q3–Q1) from the T15 concentration in the Sling after Habituation (IQR = 1.08) appeared lower compared to the sling (IQR = 3.15) and V-bench (IQR = 2.38). This finding should be interpreted with caution due to the heterogeneous group of animals, but it could indicate that habituation to the sling may reduce variation, which calls for further studies.

The third part of the study aimed to demonstrate that a force-free alternative, namely clicker training, is available. The first two parts of the study clearly demonstrated that the minipigs reacted negatively when they were restrained and their ability to escape the unpleasant scenario was eliminated. As a means to avoid using forceful handling and restraint, 21 days of positive reinforcement/clicker training were added, and as a result, it was indeed possible to draw jugular blood from two pigs, namely gilts 3 and 5. It was also possible to insert the needle in boar 3 without any reactions from the pig. However, the technician failed to locate the jugular vein. This boar had previously been used to train staff in jugular vein blood sampling in a V-bench and/or sling, which might have resulted in scar tissue making the procedure more difficult. Boars number 2 and 4 did not get a fair chance of success as one or more gilts in the animal room were in estrus during the training period. This functional reinforcer (“through-bars” in contact with gilts in estrus) was, not surprisingly, a much stronger reinforcer than apple juice. The two boars were quick learners when training took place inside their pen. The training outside their pen would most likely have been successful if these boars had been housed in gender-segregated stables.

Gilt 6 needed more time to successfully allow a restraining-free blood sampling as she accidentally fell down the ramp on training day 14. It took several days to overcome the resulting fear of the ramp and continue the training. However, the trainer could guide her up the ramp and halfway into the wagon on day 21, indicating that she could have learned the final behaviour if given more time. This challenge could have been avoided if the ramp had been constructed with protective side panels. During the training sessions, the pigs could always choose to end the session by returning to their pen; however, none of the pigs chose to end the training prematurely. They all chose to work with their trainer when given the possibility, suggesting that clicker training also functions as an enrichment. We only performed the full voluntary blood sampling on day 52. More studies should be conducted to investigate how many times/days in a row a clicker-trained pig will cooperate and to what extent it is necessary to maintain the training without performing the actual blood sampling. A limiting factor in the clicker training part was time. It is often argued that clicker training is impractical and economically unfeasible because several weeks and perhaps months may be spent to clicker train a minipig for cooperation in a study that only lasts, for example, three weeks. In this study, however, we have demonstrated that the necessary training for blood sampling can be performed in three weeks. In this study, the housing facility, the surrounding environment and the design of the set-up were not optimal and if these factors were improved the training success would most likely be increased. The minipigs in this study received 21 days of clicker training, equivalent to a standard acclimatisation and socialisation period in the facility [51,52]. Each minipig received about 60 min of CT in total. A typical socialisation period in this research facility involves shorter daily sessions totalling about 30 min per minipig over a 14-day period. While CT requires time to educate skilled animal trainers, it eliminates the need for technicians to wear hearing protection and lift 20–30 kgs minipigs during a blood sampling procedure. Additionally, once minipigs are trained to participate willingly in blood sampling, they are also trained to follow a target stick. This skill is useful for various other tasks, such as weighing, clinical observations, and relocation to different stables in the facility. Therefore, the time invested in CT may more often than not be recuperated during a study. Compared to the two traditional methods for blood sampling—the V-bench and the sling—training the pigs to voluntarily accept the blood sampling procedure by use of clicker training is obviously a better alternative. Increased awareness of environmental factors and a higher level of willingness from management and researchers to allocate the necessary resources are necessary to obtain the experience and knowledge necessary to identify how the training environment can be designed to maximise the success rate.

## 5. Conclusions

When we work with laboratory animals, it is our duty to ensure their well-being and safety—both to optimise animal welfare, but also to ensure that the resulting data are both reliable, robust and translatable, so excessive and unnecessary use of animals due to poorly designed research is avoided. Our study suggests discontinuing the use of V-benches for blood sampling in minipigs and using slings or clicker training instead. Whether habituation to the sling will add positively to animal welfare remains to be shown. Training the pigs to voluntarily accept blood sampling is undoubtedly the optimal way to obtain blood samples from the jugular vein in minipigs.

## Figures and Tables

**Table 1:** Animal ID (number), sex, age, bodyweight and previous experiences in the research facility.

Animal ID	1	2	3	4	5	6
Sex	Boar	Boar	Gilt	Boar	Gilt	Gilt
Date of birth	26.10.22	22.02.23	22.12.22	25.10.22	28.01.23	10.02.23
Weight (kg)	21.6	14.4	21.4	22.6	15.1	15.8
OD	4 x	---	4 x	---	**	---
ID	1 x	---	---	---	**	---
Capsule dosing	---	---	---	1 x	**	---
IM	1 x	---	2 x	2 x	**	---
OA	1 x	---	---	---	**	---
BS, V-bench	1 x	---	1 x	3 x	**	---
BS, sling	---	---	---	---	**	---

x = a specific day (i.e., 4 x equals four days). Each pig was typically used 2–4 times a day to train a particular technique. Animal number 5 was dosed in a previous study at Scantox A/S and information regarding the included procedures was not available, as they were classified (**). OD = Oral dosing (oral gavage); ID = intradermal injection; IM = intramuscular injection; OA = Ophthalmic administration; BS = blood sampling.

**Table 2:** The two groups and the restraining methods on their respective test days.

Group	Animal (ID No; Sex; Bodyweight)	Test Day 1	Test Day 2	Test Day 4	Test Day 5	Test Day 15	Test Day 16
1	1; boar; 21.6 kg	V-bench		Sling		Sling	
	2; boar; 14.4 kg	Sling		V-bench		Sling	
	3; gilt; 21.4 kg	V-bench		Sling		Sling	
2	4; boar; 22.6 kg		Sling		V-bench		Sling
	5; gilt, 15.1 kg		V-bench		Sling		Sling
	6; gilt; 15.8 kg		V-bench		Sling		Sling

**Table 3:** The two behaviours documented during simulated blood sampling in the V-bench and sling. Gray areas symbolises that no testing was done.

Behaviour	Description
Vocalisation	High frequency, open-mouth vocalisation (screams, squeals) during the procedure (from capturing in pen until the pig is released back in the pen). Oinks/grunts were not scored.
Struggle	Escape-related behaviour. Struggling with the head and/or legs during the procedure (from capturing in pen until the pig is released back in the pen).

**Table 4:** The behaviour before (B), during (D), and after (A) simulated blood sampling in V-bench and sling, respectively.

		V-Bench		Sling	
Animal ID	Sex	Vocalising	Struggling	Vocalising	Struggling
1	Boar	B D	-	B	-
2	Boar	B D	-	B D	-
3	Gilt	B D A	A	B D A	D A
4	Boar	B D A	D	B D A	D A
5	Gilt	B D A	B D A	B D A	B D A
6	Gilt	B D A	A	B A	-

## Data Availability

Dataset available on request from the authors.

## Supplementary Materials

The following supporting information can be downloaded at: https://www.mdpi.com/article/10.3390/ani15030407/s1, Figure S1: The initial behavioural assessment done prior to study start.; Figure S2: Mini pigs in V-bench and sling; Table S1: Training protocol with the 13 steps and relevant criteria; Video S1: Force-free blood sampling (https://zenodo.org/uploads/14512168?token=eyJhbGciOiJIUzUxMiJ9.eyJpZCI6Ijg0Mjc4MGU1LWM4ZTUtNDQzOS04MGY5LTU1ODk5YzE0NWZiYSIsImRhdGEiOnt9LCJyYW5kb20iOiIwODA4YWNlMGVkMjgyNmJiYjMwZWQzNDQ3NzUzYzczNCJ9.1lGqQY6bEFHz4y9RpTcuU20HtZSBgXnJWtDTHLIiq08hO24yquB8B0hzx97QvANyKDl0RIhbEumG-BkNuLhMzg (accessed on 18 December 2024)).
