# Supplementary material for: Blood Sampling in Göttingen Minipigs—A Case Study of Two Standard Methods and Clicker Training as a Restraint-Free Alternative
Source: Animals (Basel). 2025 Feb 1;15(3):407. doi: 10.3390/ani15030407 (PMC11816219; doi:10.3390/ani15030407)
Supplement: Supplementary file 1 [file animals-15-00407-s001.zip › Figure S1 The initial behavioural assessment.pdf]

Figure S1: The initial behavioural assessment done prior to study start.

NO: Latency (seconds) to approach a novel object (an unknown object; a bucket).
HAT (Human approach test): Latency (seconds) to approach an unknown person.
HIT (Human interaction test): Latency to acceptance of being touched behind their ears by the unknown person.

**Animal ID**	**Sex**	**Behavior Assessment (latency, seconds)**		
		**NO**	**HAT**	**HIT**
**1**	Boar	0	18	5
**2**	Boar	0	0	0
**3**	Gilt	0	0	0
**4**	Boar	0	2	2
**5**	Gilt	0	22	5
**6**	Gilt	0	25	7
