# Supplementary material for: Blood Sampling in Göttingen Minipigs—A Case Study of Two Standard Methods and Clicker Training as a Restraint-Free Alternative
Source: Animals (Basel). 2025 Feb 1;15(3):407. doi: 10.3390/ani15030407 (PMC11816219; doi:10.3390/ani15030407)

Figure S2. Mini pigs in V-bench and sling

Simulated blood sampling on minipigs in v-bench (left) and sling (right).

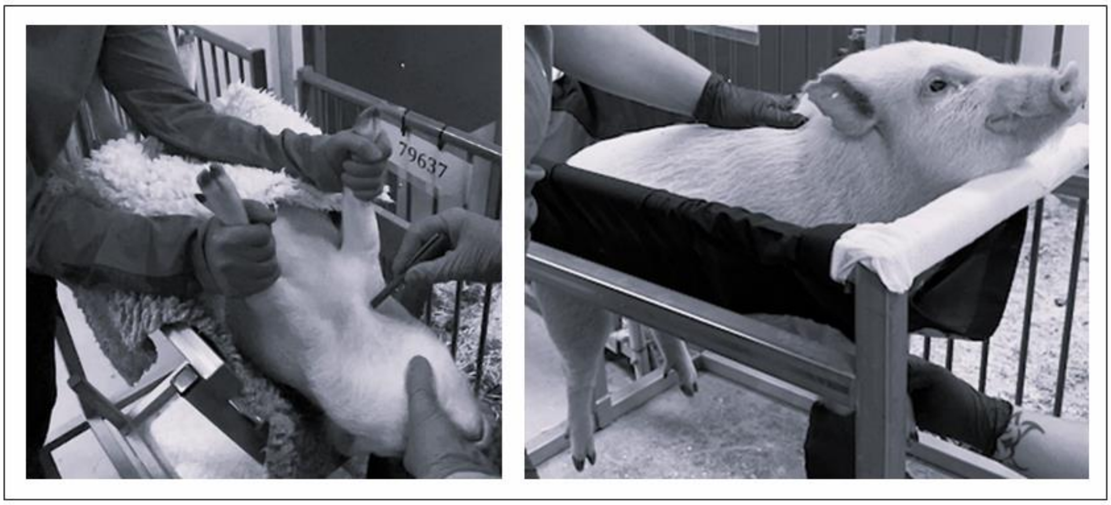

Supplement: Supplementary file 1 [file animals-15-00407-s001.zip › Figure S2 Minipigs in V-bench and sling.pdf]
