# Supplementary material for: Blood Sampling in Göttingen Minipigs—A Case Study of Two Standard Methods and Clicker Training as a Restraint-Free Alternative
Source: Animals (Basel). 2025 Feb 1;15(3):407. doi: 10.3390/ani15030407 (PMC11816219; doi:10.3390/ani15030407)
Supplement: Supplementary file 1 [file animals-15-00407-s001.zip › Table S1 Training protocol with the 13 steps and relevant criteria.pdf]

Table S1 Training protocol with the 13 steps and relevant criteria. For each pig it is indicated which steps were completed within the set time (21 days).

X (green): Criteria met.

A (blue): Criteria not reached due to environmental disturbances (gilts in estrus in the facility).

B (Yellow): Criteria not met. Fell off the ramp.

X/C (red): Boar 1 did met criteria, but the technician could not find the jugular vein. After three attempts accepted by pig 1, we did not ask for more.

Step 9-10 and 12-13: Apple juice is continuously offered to the pig, when the pig is standing calmly on station. Due to time constraints, it was decided not to train duration on station; i.e. teaching the pigs to stand still in the wagon with their snout on station waiting for the "click" and the apple juice.

STEP	APPROXIMATION/CRITERIA	Pig 1 Boar	Pig 2 Boar	Pig 3 Gilt	Pig 4 Boar	Pig 5 Gilt	Pig 6 Gilt
**1**	The click is associated with apple juice	X	X	X	X	X	X
**2**	Follows the target stick (TS) inside the pen for 4 meters.	X	X	X	X	X	X
**3**	Stands calmly with the snout into a station drinking apple juice	X	X	X	X	X	X
**4**	Follows the TS outside of the pen.	X	A	X	A	X	X
**5**	Follows the TS into a new room, in the back of the stable.	X	A	X	A	X	X
**6**	Follows the TS onto a ramp placed on the ground in the room (no wagon; ramp is horizontal).	X	A	X	A	X	X

**7**	Follows the TS into the metal frame of the wagon, with sides attached, but still placed on the ground in the room (no ramp).	X	A	X	A	X	X
**8**	Follows the TS onto the known ramp and into the known metal frame of the wagon, placed on the ground in the room.	X	A	X	A	X	X
**9**	Follows the TS onto the ramp and into the metal frame of the wagon with both sides and the station (from step 3) attached. Apple juice is continuously delivered at the station.	X	A	X	A	X	X
**10**	Follows the TS onto the ramp, up, and into the fully assembled wagon, placed approximately 30 cm above the ground on a lift, and places the snout in the station. Apple juice is continuously delivered at the station.	X	A	X	A	X	X
**11**	Stands calmly while a technician touched the ventral part of their neck using the verbal signal "touch" in their home pen.	X	X	X	X	X	X
**12**	Follows the TS onto the ramp, up and into the wagon, places the snout in the station, and stands calmly while a technician used the signal "touch" and simulates the blood sampling procedure in the external jugular vein. Apple juice is continuously delivered at the station.	X	A	X	A	X	B
**13**	Follows the TS onto a ramp, up and into the wagon, places the snout in the station, and stands calmly while a technician first uses the signal "touch" and thereafter uses the signal "pinch" followed by drawing blood from the external jugular vein. Apple juice is continuously delivered at the station.	X/C	A	X	A	X	B
